# Particle Size Effect of Lanthanum-Modified Bismuth Titanate Ceramics on Ferroelectric Effect for Energy Harvesting

**DOI:** 10.1186/s11671-021-03567-2

**Published:** 2021-07-06

**Authors:** Sangmo Kim, Thi My Huyen Nguyen, Rui He, Chung Wung Bark

**Affiliations:** 1 School of Intelligent Mechatronics Engineering, Sejong University, Gwangjin-gu, Seoul, 05006 Republic of Korea; 2 Department of Electrical Engineering, Gachon University, Seongnam-si, Gyeonggi-do, 13120 Republic of Korea

**Keywords:** Poly(vinylidene fluoride), BLT, Particle size, Piezoelectric nanogenerator

## Abstract

Piezoelectric nanogenerators (PNGs) have been studied as renewable energy sources. PNGs consisting of organic piezoelectric materials such as poly(vinylidene fluoride) (PVDF) containing oxide complex powder have attracted much attention for their stretchable and high-performance energy conversion. In this study, we prepared a PNG combined with PVDF and lanthanum-modified bismuth titanate (Bi_4−X_La_X_Ti_3_O_12_, BLT) ceramics as representative ferroelectric materials. The inserted BLT powder was treated by high-speed ball milling and its particle size reduced to the nanoscale. We also investigated the effect of particle size on the energy-harvesting performance of PNG without polling. As a result, nano-sized powder has a much larger surface area than micro-sized powder and is uniformly distributed inside the PNG. Moreover, nano-sized powder-mixed PNG generated higher power energy (> 4 times) than the PNG inserted micro-sized powder.

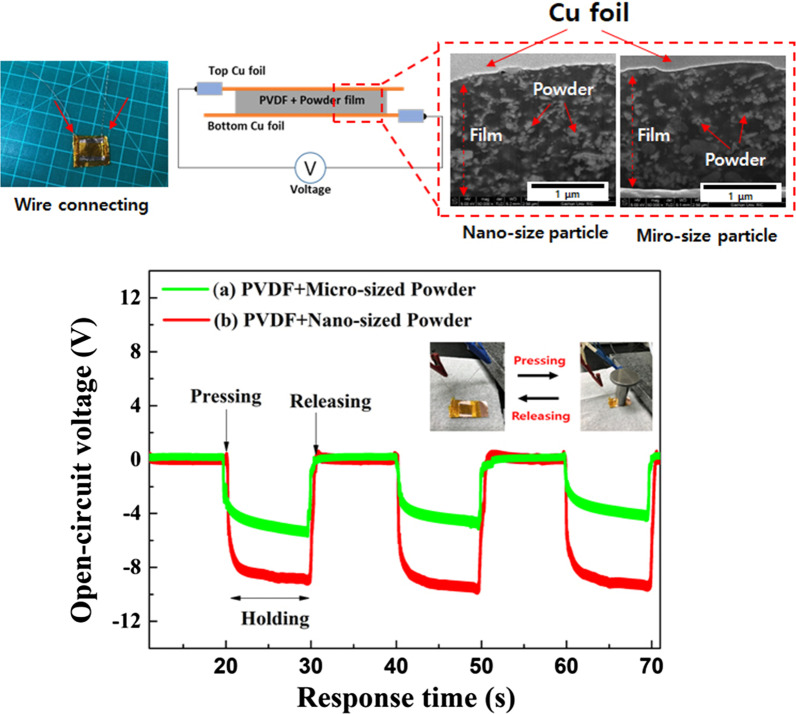

## Introduction

Energy harvesting is a promising energy-saving technology that enables us to live on Earth continuously. Energy harvesting has attracted much attention for enabling the stable operation of Internet of Things (IoT) applications. A high energy-harvesting performance is a key to how much relatively small energy and power can be collected. Moreover, stretchable and wearable functions are required for all state-of-the-art devices [1–3]. Energy harvesting technologies for collecting energy sources, which originate from mechanical pressing, vibrations (piezoelectric), temperature gradient (thermoelectric), and solar light (photovoltaic), have rapidly developed over the past decade; these involve the process of capturing energy from one or more renewable energy sources and converting it into usable electrical energy [4–6].

The piezoelectric technique has been most commonly used among various techniques because of its transduction simplicity and relative implementation ease in various application fields. Piezoelectric nanogenerator (PNG) systems include two system types: piezoelectric ceramics and piezoelectric polymer-based organic piezoelectric materials [7, 8]. Piezoelectric ceramics have a high energy-collection ability. However, they do not easily bend and are easily broken by mechanical shock. Compared to piezoelectric ceramics, piezoelectric polymers are stronger than breaking and bending polymers. Piezoelectric polymers have been fabricated using organic piezoelectric materials. Poly(vinylidene fluoride) (PVDF) was introduced, leading to PNG with polymers [9, 10]. Several attempts have been made to incorporate ceramic and organic materials inside the polymer matrix by changing the device structure to improve the energy-harvesting performance of piezoelectric polymers [11–13]. Moreover, for high-performance in the device, it has been introduced that surface treatment or controlling the particle size and shape for large surface area. [14–16].

In this study, we selected lanthanum-modified bismuth titanate (BLT, Bi_3.25_La_0.75_Ti_3_O_12_) ceramics, which have been reported as suitable insulators with strong sustainability, low processing temperature, and large values of remnant polarization [17, 18]. BLT has come from lanthanum (La)-doped Bi_4_Ti_3_O_12_ (BTO) which is representative of ABO_3_ perovskite compounds which belongs to Aurivillius phases. Instead of Bi ions near the Ti–O octahedron layers in BTO, La ion doping could improve its physical properties and crystallinity by decreasing oxygen vacancies and crystalline structure defects [19, 20]. First, nano-sized BLT powder was prepared via high-energy ball milling from a micro-sized powder [21]. As the particle size decreased, the surface area of the nano-sized particles improved up to 10 times that of the micro-side. Then, we synthesized PNG with a combination of ferroelectric materials to improve its energy-harvesting performance and the effect on the particle size of BLT ceramics (micro- and nano-) contained in PNG devices without polling. Compared to the PNG inserted micro-sized powder, we observed that the energy generation performance was improved by more than four times compared to nano-sized powder-mixed PNG.

## Methods

### Starting Chemical Materials

As starting chemical materials for BLT, Bi_2_O_3_ (< 99.9%), TiO_2_ (< 99.99%), La_2_O_3_ (< 99.99%), and binary oxide powders were purchased from Kojundo Chemical Company. PVDF powder and N, N-Dimethylacetamide (DMA), and ethanol were purchased from the Sigma-Aldrich Chemical Company. All chemical materials and solvents were used without further purification during the experimental procedure.

### Sample Fabrications

**Fig. 1:**
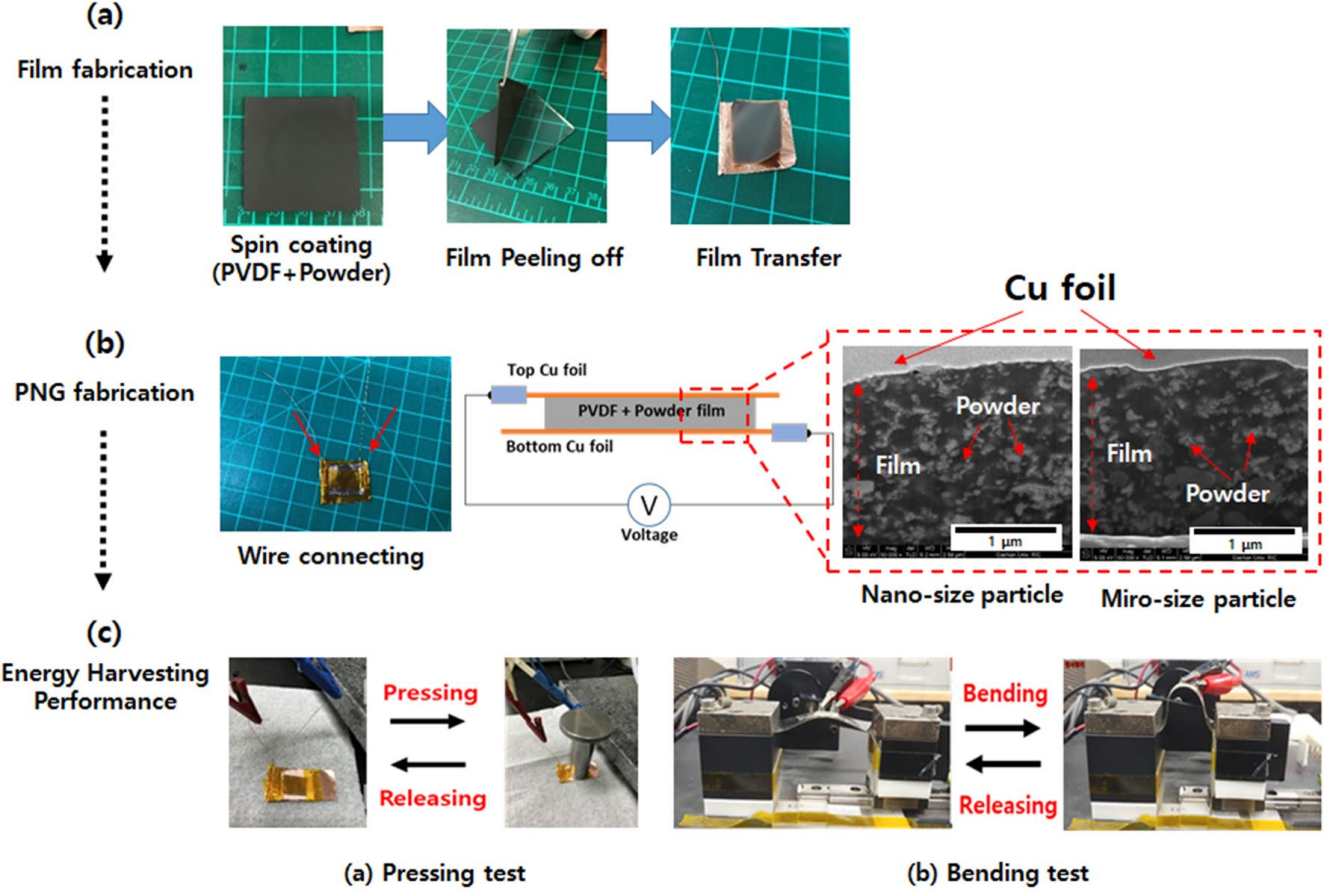
Fabrication process of PNG contained PVDF and BLT powder

We fabricated a PNG using the following three steps: **Step 1**. **Synthesis of BLT powder:** Based on our previous reports [22, 23], we prepared BLT powder with micro-sized particles. The as-prepared BLT oxide composite powders were Bi_3.25_La_0.75_Ti_3_O_12_. First, the starting chemical reagents, ethanol, and zirconia grinding beads were transferred to a Teflon bottle and thoroughly mixed in a mechanical ball mill (200 rpm) for 24 h. After the mixture was completely dried in an oven (80 °C), the obtained mixtures were calcined at 850 °C for 3 h (5.2 °C/min). **Step 2**. **Controlling particle size after calcination:** The bulk powder (micro-sized) was treated in a high-energy ball milling system (Model UAM-015, Kotobuki) with ethanol and zirconia beads (Ø < 0.1 mm). Before sample treatment, we dispersed BLT powder (3 g) and zirconia beads (400 g) in ethanol (500 mL); the mixing solution was supplied to a 0.15-L vessel. The ball milling process was carried out in the vessel at a rotor speed of 40 Hz, corresponding to 3315 rpm, for 3 h**. Step 3. Fabrication of PNG with BLT powder:** PVDF and BLT powder (40 wt.%) was dispersed in the DMA solution and stirred for 2 h at room temperature. The mixing solution was dropped on a soda-lime glass substrate and spun at 1000 rpm for 30 s. After the spin coating process, the samples were transferred to a hot plate and dried at 60 °C for 3 h to evaporate the solvent. The PVDF and BLT films were obtained by peeling the glass substrate. PNG devices were assembled with films inserted between sandwiched copper foils as the top and bottom electrodes. The detailed procedure conditions are shown in Fig. 1.

### Measurements

The morphological and microstructural properties of the synthesized micro- and nano-sized BLT powders were observed by field emission scanning electron microscopy (FE-SEM, Hitachi, S-4700) and Transmission Electron Microscope (TEM, JEOL LTD, JEM-2100F HR). The powder particle size and shape distributions in the PVDF-powder complex films in the PNG device were observed using a focused ion beam system (FIB, Nova Nano, SEM200). Nitrogen adsorption–desorption measurements were performed on a BET surface analyzer, and the specific surface areas and pore volumes were calculated using the Brunauer–Emmett–Teller (BET, Micromeritics, ASAP 2020) method. The pore size distribution of the powder was estimated from the adsorption branches of isotherms using the Barrett–Joyner–Halenda (BJH) method. The generated voltage and current were measured using an IV solution system (source meter, Keithley 2410).

### Piezoelectric Property Test for Energy Generation Performance

To evaluate the energy generation properties of the samples, we used two different types of modes: (1) pressing/unpressing and (2) bending/releasing, as shown in the energy-harvesting performance of Fig. 1c. First, the voltage generation of the samples was measured by pressing and unpressing the weight (area: 1.77 cm^2^, 194 g) in the middle of the PNG device. Then, the samples (PNG devices) were loaded on the polycarbonate substrate and connected to the IV solution equipment. For sample bending at a speed of 1 time/s, we measured the generated current and voltage of the prepared PNG devices with and without BLT powder.

## Results and discussions

### Tuning the Particle Size of BLT Ceramic and PVDF

**Fig. 2:**
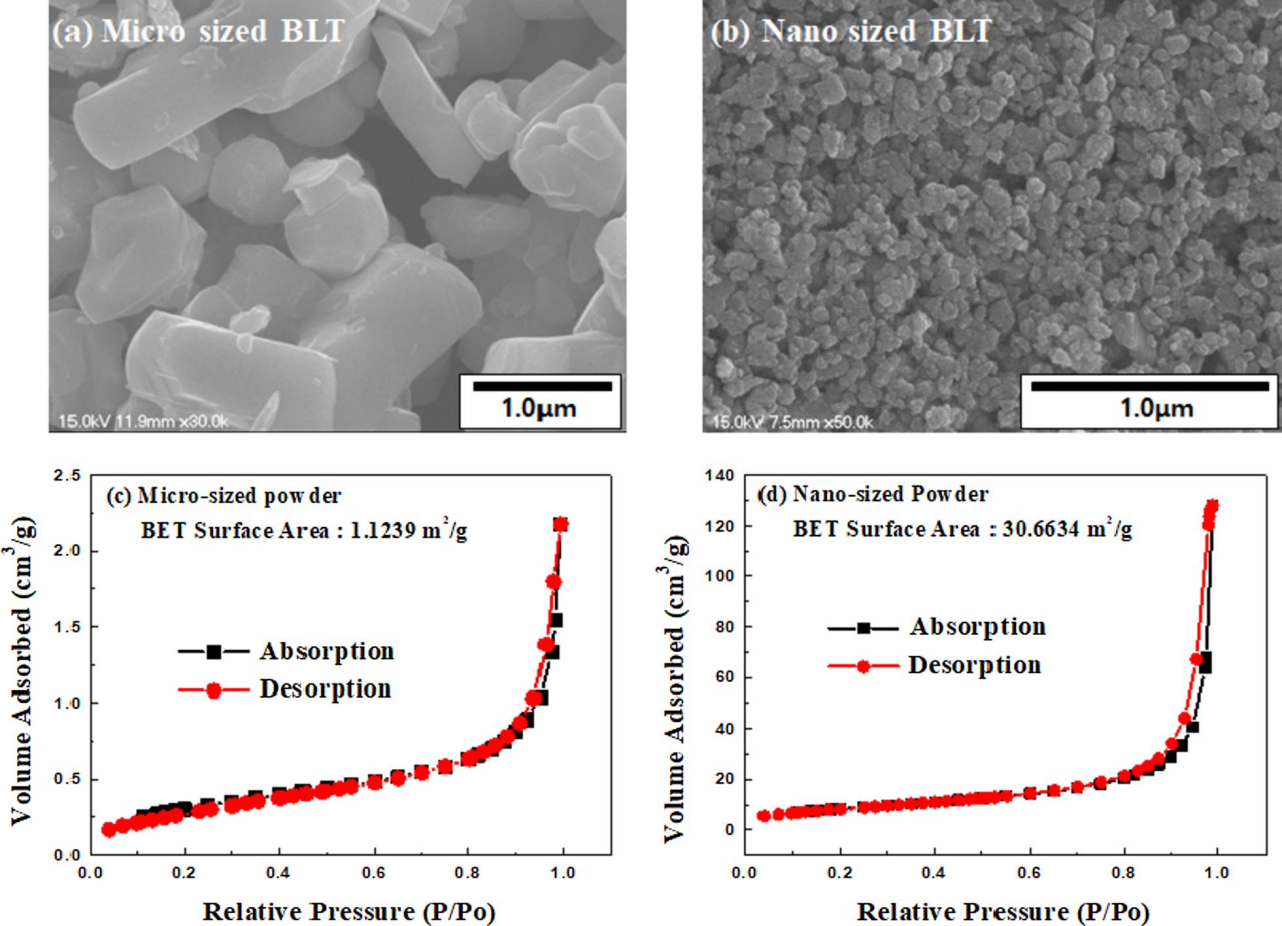
FE-SEM images and Nitrogen adsorption–desorption isotherm and pore size distribution of BLT ceramics prepared and treated by high energy ball milling for 3 h; **a** and **c** micro-sized, and **b** and **d** nano-sized powder

After the BLT powder was subjected to high-energy ball milling, the powder’s particle size became smaller and narrower. As shown in Fig. 2, the BET surface area of the powder increased from 1.5297 to 33.8305 m^2^/g. The surface area of the samples was dependent on their particle size. Figure 2c and d show the samples’ nitrogen adsorption/desorption isotherm (BJH, BET). The BET surface areas of micro-sized and nano-sized samples are approximately 1.12 and 30.67 m^2^/g, respectively. The isotherm profiles of all samples belong to type II with an H3 hysteresis loop with a pronounced hysteresis loop according to the IUPAC classification [24, 25]. This type of isotherm at low relative pressure indicates that the samples have non-porous surfaces.

**Fig. 3:**
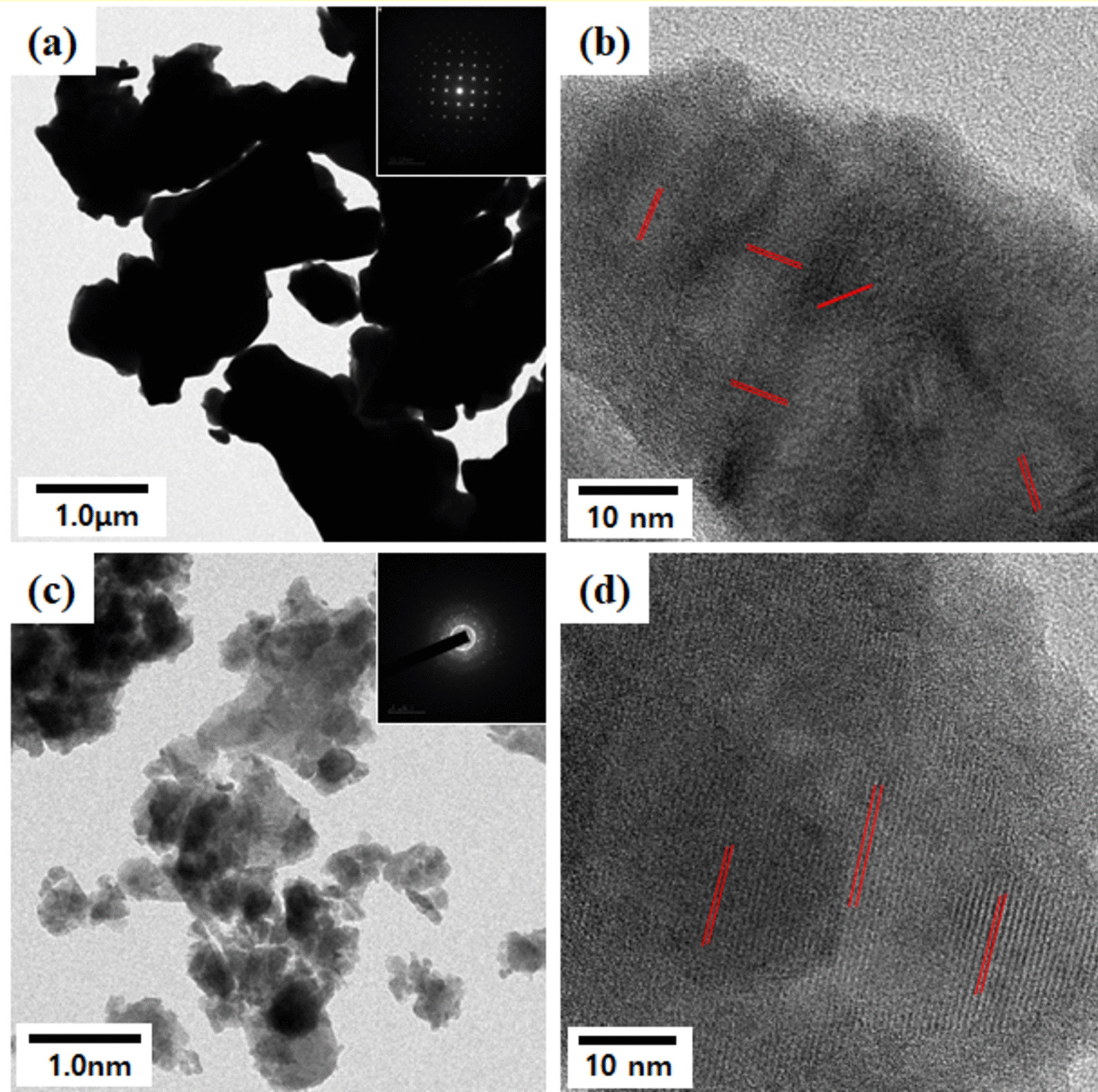
TEM images of BLT powder after and before treatment by high-energy ball milling; **a** and **b** Micro-size and **c** and **d** Nano-sized

It can be clearly seen from Fig. 2a that the morphology of micro-sized powder is mainly dominated by the presence of irregular edges grains with randomly sized polygon. We observed that the particle size decreased from the FE-SEM images in Fig. 2b after high-energy ball milling. As expected from FE-SEM and BET in Fig. 2, the observation from TEM can be confirmed that the particle size and shape were changed in a series of samples from Fig. 3. Compared to micro-sized BLT powder with microstructure, Nano-sized BLT powder is composed of grains with a size less than 100 nm. Therefore, BLT powder with Nano-sized particle may be of larger specific surface area which may be quite helpful for formal distribution for improving energy generation in NG device [21, 26].

**Fig. 4:**
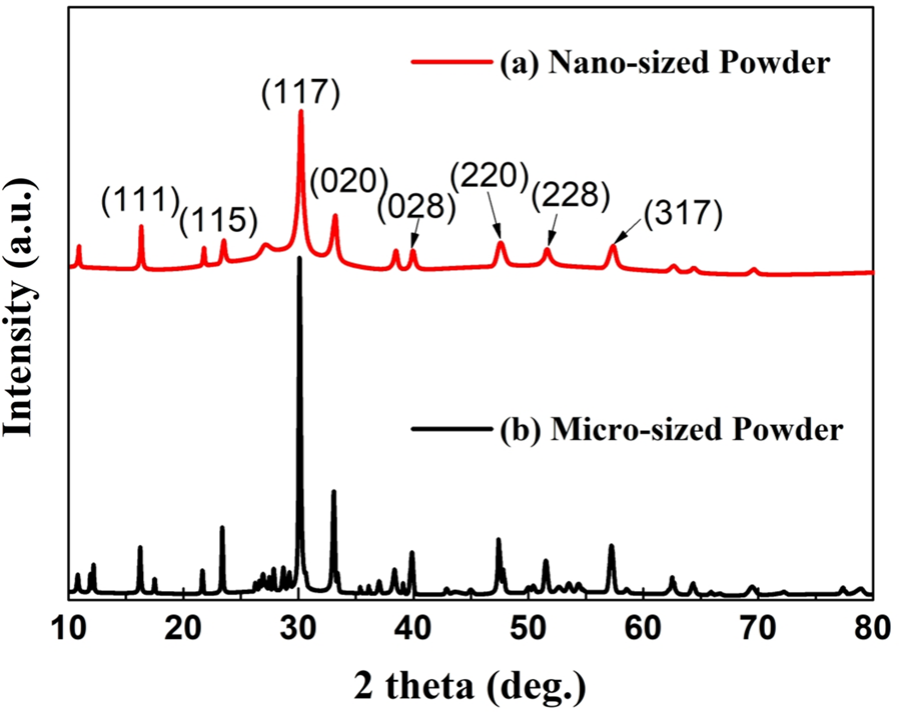
X-ray diffraction (XRD) patterns of powders: **a** nano-sized BLT powder and **b** Micro-sized BLT powder

The particle size effect in the powder is shown in the XRD patterns in Fig. 4. The XRD peaks of the powders were well matched with the standard peaks of the orthorhombic structure, although the particle size of the BLT powder decreased. Compared to the sharp diffraction lines with the micro-sized powder’s high intensity, that of nano-sized powder was broadened considerably with lower intensity owing to the increased internal lattice strain during ball milling [23, 26]. We confirmed that the Aurivillius structure indexed assuming significant peaks (117), (020), and (208) were maintained without breaking symmetry despite the particle size change by high-energy ball milling. The crystallite size of the products was determined from the most significant peaks (117) in the XRD patterns according to Scherrer’s equation, *D* = *Kλ*/*β*cos*θ*, where *λ* is the X-ray wavelength (1.54056 Å), β is the full width at half maximum (FWHM), θ is the Bragg angle, and *K* is the shape factor [27]. Their crystallite size was ~ 50 nm. We observed that nano-sized particles were uniformly distributed in the powder, as shown in Fig. 2b.

**Fig. 5:**
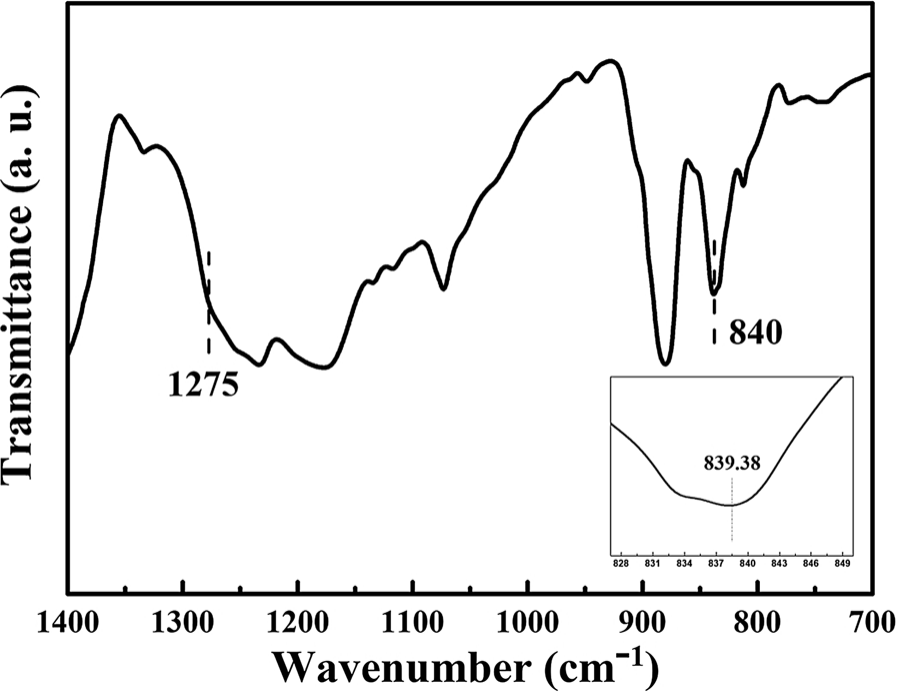
Fourier-transform infrared (FT-IR) spectrometer of prepared pure PVDF film

Pure PVDF is known for its *α*, *β*, and *γ* crystalline phases [28]. To generate piezoelectricity, conventional PVDF should be formed in the *β*-phase. We investigated whether β-PVDF was formed using a FT-IR spectrometer, as shown in Fig. 5. From the detected peaks located at 1275 and 840, we concluded that the two bands were attributed to β-PVDF.

In addition, to generate piezoelectricity, PVDF film has to acquire a high polarity that depends on the arrangement of (–CH_2_CF_2_–) units in the material. In general, β-phase PVDF (β-PVDF) exhibits the best polarity among three crystalline phases as β-PVDF possessed all the poles in the same direction. FTIR spectrum of the PVDF film in the range 1400 to 700 cm^−1^ is shown in Fig. 5. The result exhibits almost characteristic peaks of PVDF. It is clear that the exclusive peaks at 1275 (CF_2_ bending) and 840 cm^−1^ (CH_2_ rocking) indicated a dominant of β-phase [29–31]. It is worth noting that β-PVDF film would be formed at a moderate temperature accompanied by slow evaporation of the solvent. Hence, the pure and doped-BLT particles PVDF films would slowly evaporate on the hot plate at 60 °C to create the PNGs.

### Energy generation performance of PNG with PVDF and BLT powder

**Fig. 6:**
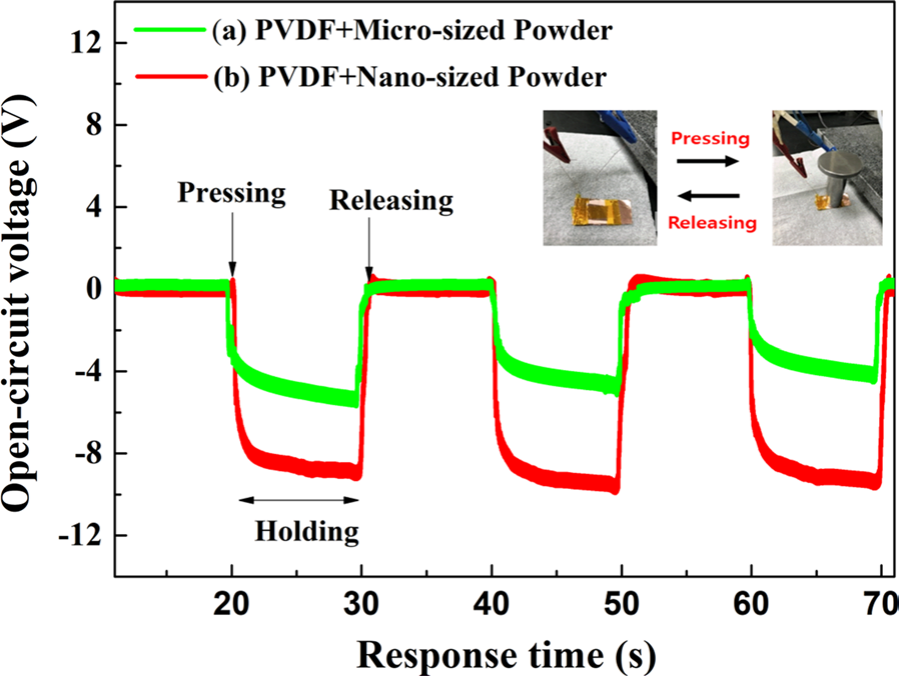
Open-circuit voltage generation of PNG with PVDF and Powder during pressing and releasing conditions

To confirm the powder particle size effect in the PNG, we measured the voltage generation performance without high electric field poling treatment. Figure 6 shows the voltage generation property of the prepared PNG with PVDF and BLT powders (micro- and nano-sized). We calculated the energy-producing part (active area) in contact with the weight in the PNG device (Device size: 3.0 cm × 3.0 cm); the active area of the PNG was 4 cm^2^. The generated voltage was recorded for 20 s of pressing and unpressing with 3 N.

**Fig. 7:**
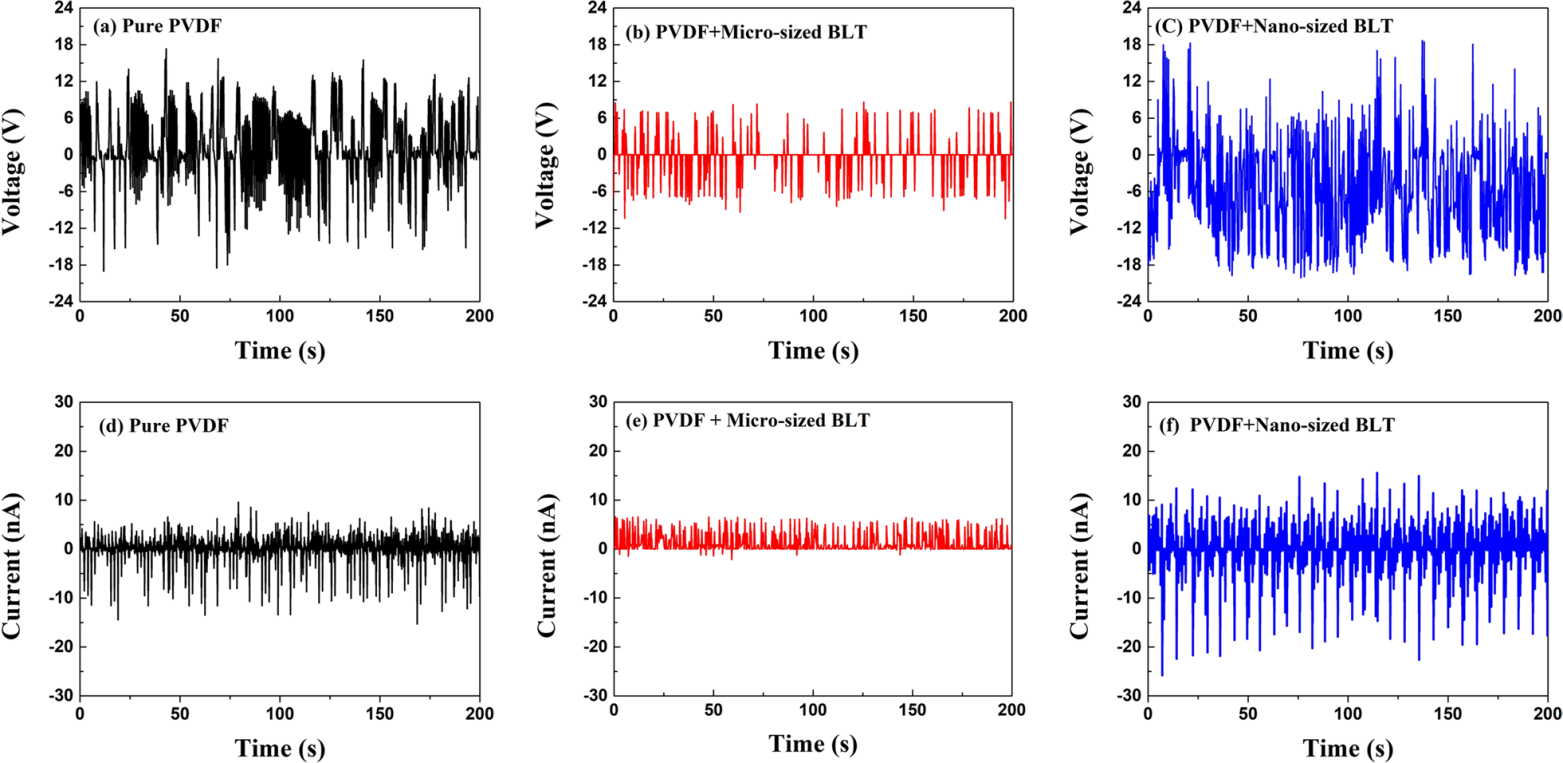
Open-circuit voltage and the current generation of pure PVDF and PVDF/Powder composites during bending connected to IV solution equipment

When vertical stress is applied to the samples by pressing up and down, the piezoelectric potential energy is generated in one side direction. The piezoelectricity direction of pure PVDF is negative along the vertical axis with polarity. Regardless of particle size, the PNG device prepared with the PVDF and BLT powders had a positive voltage along with the polarity of pure PVDF. After pressing the samples, the output voltage was observed as the content increased with the particle size of the BLT powders. Moreover, the PNG output voltage was reduced with powder (micro-sized), where the output voltage decreased with pressing, suggesting that the particle size of the powder is required to obtain the maximum output performance of the nanogenerator. Compared to the micro-sized powder, the nano-sized powder may be disturbed in the film. Among all the samples, the nano-sized PNG had the highest open-circuit voltage of 10 *V*_pk-pk_. After the stressing force was applied to the samples, the PNG samples maintained energy production for approximately 20 s, because the BLT powder was present inside the PNG. We assumed that a recovery time of 10 s is required to return to the zero level (Fig. 7).

## Conclusions

We prepared PNG devices with organic–inorganic composites containing PVDF and BLT. All PNGs were tested under two different energy harvesting performances (pressing and bending) to investigate the piezoelectric performance of the PNG without high electric field poling. Compared to pure PVDF, the micro-sized powder-inserted PVDF exhibited lower energy generation properties. However, by decreasing the particle size in the powder, we confirmed that the piezoelectric performance was improved by a factor of four owing to its large surface area and uniform distribution in PNG devices.

## Data Availability

All data supporting the conclusions of this article are included within the article.

## Abbreviations

PNG: Piezoelectric nanogenerator
BLT: Lanthanum-modified bismuth titanate (Bi_4−X_La_X_Ti_3_O_12_)
PVDF: Poly(vinylidene fluoride)
FE-SEM: Field emission scanning electron microscopy
TEM: Transmission electron microscope
XRD: X-ray diffraction
FWHM: Full width at half maximum
FT-IR: Fourier-transform infrared
